# Multimodal data-driven approaches in retinal vein occlusion: A narrative review integrating machine learning and bioinformatics

**DOI:** 10.1016/j.aopr.2025.07.002

**Published:** 2025-07-14

**Authors:** Chunlan Liang, Lian Liu, Jingxiang Zhong

**Affiliations:** Department of Ophthalmology, The First Affiliated Hospital of Jinan University, Guangzhou, China

**Keywords:** Bioinformatics, Clinical prediction models, Deep learning, Markers, Multimodal data, Machine learning, Retinal vein occlusion

## Abstract

**Background:**

Retinal vein occlusion (RVO) is a leading cause of visual impairment on a global scale. Its pathological mechanisms involve a complex interplay of vascular obstruction, ischemia, and secondary inflammatory responses. Recent interdisciplinary advances, underpinned by the integration of multimodal data, have established a new paradigm for unraveling the pathophysiological mechanisms of RVO, enabling early diagnosis and personalized treatment strategies.

**Main text:**

This review critically synthesizes recent progress at the intersection of machine learning, bioinformatics, and clinical medicine, focusing on developing predictive models and deep analysis, exploring molecular mechanisms, and identifying markers associated with RVO. By bridging technological innovation with clinical needs, this review underscores the potential of data-driven strategies to advance RVO research and optimize patient care.

**Conclusions:**

Machine learning-bioinformatics integration has revolutionised RVO research through predictive modelling and mechanistic insights, particularly via deep learning-enhanced retinal imaging and multi-omics networks. Despite progress, clinical translation requires resolving data standardisation inconsistencies and model generalizability limitations. Establishing multicentre validation frameworks and interpretable AI tools, coupled with patient-focused data platforms through cross-disciplinary collaboration, could enable precision interventions to optimally preserve vision.

## Graphical abstract

**Figure:**
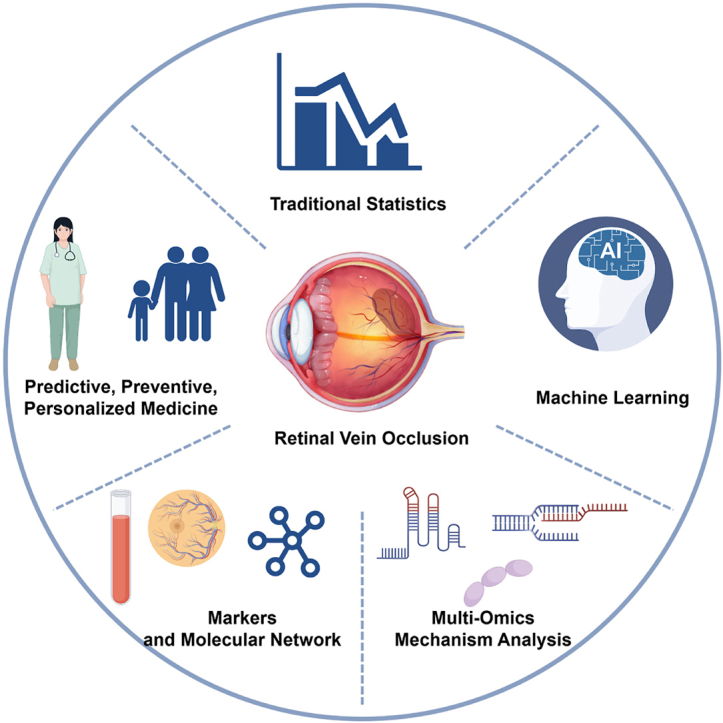

## 1 Introduction

Retinal vein occlusion (RVO) is a major global cause of vision loss, with a 2015 worldwide prevalence of 0.77% (affecting approximately 28.06 million people aged 30–89 years), imposing a significant healthcare burden.^1^ The pathophysiology of RVO involves intricate interactions among vascular obstruction, ischemic damage, and inflammatory cascades, which collectively contribute to vision loss and diminished quality of life.^2^ Current clinical management remains predominantly reactive, focusing on mitigating symptoms after their onset rather than addressing underlying mechanisms or preempting disease progression.

Recent advances in machine learning (ML) and bioinformatics have introduced transformative tools for analyzing RVO's complexity.^3^ The integration of multimodal data—spanning clinical records, retinal imaging, and molecular profiles—has enabled the development of computational models capable of identifying high-risk patients and optimizing therapeutic strategies.^4^ These innovations mark a shift toward data-driven approaches that prioritize early intervention and tailored treatments, aiming to improve clinical outcomes while reducing the socioeconomic impact of advanced disease.^5^

This review explores multimodal data-driven approaches in RVO research, focusing on three areas: (1) ML-based predictive models and deep analysis; (2) bioinformatics-driven molecular insights into potential therapeutic targets; and (3) biomarker discovery and clinical utility. By synthesizing these advancements, this work aims to guide scalable, patient-centered strategies for improving RVO management.

## 2 Method of literature search

This narrative review employed structured searches in PubMed, Web of Science, and Google Scholar (January 2010–April 2025) using combined Boolean queries of three conceptual domains: (1) RVO terminology ("Retinal Vein Occlusion"[Mesh] OR "Branch Retinal Vein Occlusion"[tiab]), (2) artificial intelligence ("Machine Learning"[Mesh] OR "Deep Learning"[tiab]), and (3) bioinformatics approaches ("Computational Biology"[Mesh] OR "Multi-Omics Integration"[tiab]). After initial identification of 527 records, titles/abstracts were evaluated against pragmatic inclusion criteria, prioritizing studies that applied multimodal data-driven strategies to RVO biomarker discovery, pathogenesis elucidation, or therapeutic development. Non-English publications were retained only when accompanied by English abstracts to capture critical methodological insights. All exclusion decisions were based on multimodal methodology relevance, not evidence level.

## 3 Machine learning in RVO: prediction models and deep analysis

The rapid development of ML and deep learning (DL) technologies is having a significant impact on the risk and prognosis prediction paradigm of RVO. Researchers have gradually shifted from traditional statistical models to intelligent algorithms driven by multimodal data, intending to construct more accurate and clinically valuable prediction tools.^6^

### 3.1 Evolution from traditional statistics to clinical prediction models

Initial risk stratification in RVO primarily relied on conventional statistical methods, notably logistic and Cox regression analyses. These approaches typically utilize demographic characteristics (*e.g.*, age, gender) and co-morbidities (*e.g.*, hypertension, diabetes).^7^^,^^8^ While effective for elucidating relationships through established hypotheses, these methods often struggle to capture complex non-linear interactions within intricate clinical datasets.^9^

Contemporary clinical prediction models overcome these limitations by leveraging ML or advanced statistical techniques. By analyzing large-scale clinical data, these approaches identify subtle patterns, effectively process heterogeneous variables, enable population-specific optimization, and enhance predictive accuracy and generalizability.^10^

Risk Prediction Models: A nomogram integrating HDL-C, neutrophil count, and hypertension history (sourced from electronic medical records [EMRs]) predicted overall RVO risk (area under the curve [AUC] ​= ​0.741; 95% CI: 0.646–0.836).^11^ Subtype-specific models demonstrated enhanced discrimination: predictors for CRVO (neutrophil-lymphocyte ratio [NLR], HDL-C, PDW, diabetes, cerebral infarction, coronary artery disease) achieved AUC ​= ​0.77 (95% CI: 0.65–0.86), while BRVO predictors (NLR, hypertension, age, BMI) yielded AUC ​= ​0.95 (95% CI: 0.89–0.98).^12^ Automated ML identified age, low-income status, and systolic blood pressure (SBP) as key determinants (AUC ​= ​0.856; 95% CI: 0.835–0.875) for RVO, enabling code-free implementation using health check-up data.^13^

Prognostic Models: Key factors predicting visual acuity outcomes after anti-VEGF therapy in macular edema (ME) secondary to RVO (BRVO-ME), such as age, baseline best-corrected visual acuity (BCVA), and ellipsoid zone (EZ) continuity (mean AUC ​= ​0.93).^14^ For RVO-ME recurrence, independent predictors comprised pretreatment central retinal thickness (CRT), disease duration, hyperreflective foci, disturbed inner retinal structure (DRIL), and injection frequency (AUC ​= ​0.939; 95% CI: 0.892–0.985).^15^

### 3.2 Technological breakthroughs in ML and DL

Recent technological advancements in ML and DL have provided a novel approach to the analysis of image features, with three key advantages being evident.

#### 3.2.1 Deep analysis of image features

Hierarchical feature extraction frameworks have advanced retinal image analysis. Residual convolutional neural networks (CNNs) demonstrate strong performance in feature representation and cross-modal synthesis for RVO diagnosis.^16^ For example, end-to-end CNNs generate non-invasive fundus fluorescein angiography (FFA) images from fundus photography, reducing reliance on fluorescent dyes while preserving vascular morphology and improving diagnostic accuracy.^17^ Further studies have shown that Xiang et al.^18^ achieved the detection of clinical stage RVO with 83.67% sensitivity and 98.10% specificity by analyzing 10,549 single-center fundus photographs using the ResNet-50 architecture. In addressing the challenge of subtype-specific modeling, the layered attention network (LA-Net) multitasking framework achieved BRVO-specific modeling through the design of a shared convolutional layer and an independent classification layer.^17^

Compared to CNNs, Swin Transformer employs attention mechanisms that focus on feature interactions beyond local receptive fields. The RVO subtype classification model based on the Swin Transformer demonstrates strong performance, with 99.98% ​± ​0.015 recognition accuracy for CRVO and 98.88% ​± ​0.08 for BRVO.^19^ Furthermore, the application of generative adversarial network (GANs) in RVO research has been extended to the joint optimization of image generation and lesion segmentation, including short-term anti-VEGF therapy efficacy prediction using synthetic ME images.^20^

#### 3.2.2 Dynamic risk warning

Longitudinal monitoring is critical for slowly progressive eye diseases. Dynamic risk warning (DRW) systems identify emerging risks by analyzing temporal data trends.^21^ In this domain, the integration of the Transformer architecture surpasses the constraints of conventional static models and facilitates the dynamic prediction of disease progression from longitudinal medical images.^22^ For instance, in the study of age-related macular degeneration (AMD) and glaucoma, fundus photography image sequences have been employed to simulate the disease's temporal evolutionary pattern and capture changes in pathological features at irregular time intervals.^23^ While RVO-specific DRW research remains limited, promising approaches include: monitoring the rate of retinal blood flow density decay through continuous optical coherence tomography angiography (OCTA) images, or integrating time-series data such as medication adherence and metabolic indexes, to construct an RVO-specific early warning model and provide a scientific basis for early intervention.

#### 3.2.3 Multi-source heterogeneous data fusion

Integrating multimodal data presents significant challenges in data science. The goal is to effectively fuse diverse sources, formats, and modalities to unlock their combined information potential, thereby enhancing decision support.^24^ Knowledge graphs offer a novel paradigm for cross-modal integration. By establishing networks of entities and relationships, they provide a structured semantic framework that facilitates information correlation and complementarity across modalities.^25^ Within ophthalmic record management, including RVO, multimodal knowledge graphs enable the integration of patient cases and diagnostic data. This integration improves medical record utility and supports clinical decision-making, advancing AI applications in healthcare.^26^

## 4 Multi-omics networks: mechanistic insights

Advances in bioinformatics have unveiled novel insights into the molecular landscape of RVO. By integrating multi-omics data (*e.g.*, transcriptome, metabolome, epigenome) and regulatory network analysis, researchers have gradually revealed the pathophysiological features, subtype heterogeneity, and potential therapeutic targets of RVO.

### 4.1 Multi-omics mechanism analysis

#### 4.1.1 Transcriptomics: gene expression and its interactions with cells

Transcriptomics captures the dynamic alterations in gene expression in RVO, bridging upstream genomic variations and downstream metabolic phenotypes. Laser-induced RVO models reveal characteristic molecular signatures in ischemic retina, encompassing upregulated hypoxia markers (Eno1, MMP9, Glut1), pro-angiogenic drivers (VEGFA, ADM, PLAUR), and inflammatory mediators (IL6, CCL2, MMP3), suggesting a coregulatory axis.^27^^,^^28^ Single-cell studies in ischemia-reperfusion models implicate Müller glia in neurovascular injury, potentially via NF-κB-mediated spatiotemporal expression of ferroptosis genes (Fth1, Ftl1, Hmox1).^29^ Spatial transcriptomics further delineates endothelial-RPE interactions (Rho GTPase/integrin) and coregulation by VEGF/TGFB1 in macular neovascularization.^30^ Critically, extending these multimodal approaches to human RVO holds promise for resolving the 3D neurovascular-inflammatory network, spatiotemporal ligand-receptor dynamics, and neovascular evolution.

#### 4.1.2 Metabolomics: energy metabolism imbalances and damage mechanisms

Metabolomics quantifies low-molecular-weight compounds as terminal functional phenotypes in RVO. The metabolically active retina employs aerobic glycolysis in photoreceptors for energy homeostasis and switches to anaerobic glycolysis during hypoxia.^31^ CRVO patients demonstrate aqueous humor (AH) metabolic disturbances: reduced glutamate (4.26 vs. 6.89 ​μg/ml; *P* ​= ​0.009) and glutamine (10.96 vs. 18.35 ​μg/ml; *P* ​= ​0.017).^32^ Furthermore, post-anti-VEGF therapy, increased lactate levels (*t* ​= ​2.273, *P* ​= ​0.045) inversely correlated with central macular thickness (CMT) reduction (r ​= ​−0.745; *P* ​= ​0.003). The retinal ganglion cell (RGC) thickness negatively correlated with elevated glutamine (r ​= ​−0.619; *P* ​= ​0.024) and glucose (r ​= ​−0.754; *P* ​= ​0.003).^32^

Elevated homocysteine levels (hyperhomocysteinemia) may contribute to the susceptibility to RVO, although study outcomes vary. Minniti et al.^33^ found that in CRVO patients, homocysteine levels were significantly higher (OR ​= ​1.20, 95% CI: 1.02–1.41; *P* ​= ​0.03), while Vitamin B12 levels were lower (OR ​= ​0.997, 95% CI: 0.99–1.0; *P* ​= ​0.008) compared to controls. In contrast, BRVO patients exhibited no significant difference in homocysteine levels.^33^ However, a meta-analysis reported a pooled OR of 0.87 (95% CI: 0.59–1.15) for RVO, which was statistically significant (*P* ​< ​0.05) in the NRVO group.^34^ Pathogenic mechanisms involve vascular endothelial injury through reactive oxygen species generation, endoplasmic reticulum stress, and impaired nitric oxide signaling.^35^ These alterations promote thrombogenesis and blood-retinal barrier compromise.^36^ B-vitamin supplementation reduces homocysteine levels by approximately 25%, which could potentially prevent progression to ischemic RVO with vision loss and facilitate early recovery.^37^^,^^38^

#### 4.1.3 Epigenetics: DNA methylation and histone modifications

Epigenetic mechanisms, including DNA methylation and histone modifications, regulate gene expression and cellular stress responses in RVO.^39^ At the level of molecular mechanisms, DNA methylation dynamics (*e.g.*, oxidative stress pathway genes and vasoregulatory genes) drive the damage cascade response by silencing protective retinal pathways.^40^ Furthermore, histone post-translational modifications (*e.g.*, acetylation/methylation balance) have been found to have a critical role in maintaining retinal ganglion cell homeostasis.^41^ Clinically, L-methylfolate supplementation lowers homocysteine via dual mechanisms – improving vascular endothelial function and enhancing DNA methyltransferase activity.^38^ This intervention correlated with restored visual acuity (≥20/25) and retinal remodeling in RVO patients,^38^ demonstrating the translational potential of epigenetic modulation.

### 4.2 Regulatory networks and molecular interactions

#### 4.2.1 MicroRNAs (miRNAs) and messenger RNA (mRNA) regulatory network

The miRNA-mRNA regulatory networks orchestrate inflammation and vascular homeostasis in RVO. By binding target mRNA 3′UTRs, miRNAs post-transcriptionally regulate gene expression.^42^ Dysregulation of specific miRNAs disrupts retinal signaling pathways.^43^ In CRVO AH, eight miRNAs (*e.g.*, miR-16, miR-20a) are significantly downregulated, inversely correlating with elevated MMP-2/9, VEGF, and IL-6 (*P* ​< ​0.01).^44^ Bioinformatics analyses verified the cascade mechanism of 'miRNA attenuation → MMP/VEGF pathway activation → retinal blood barrier disruption'.^44^ Specifically, the down-regulation of miR-16 deregulates the inhibitory effect on MMP-2/MMP-9, leading to increased extracellular matrix degradation,^45^ whereas the deletion of miR-20a leads to aberrant activation of MMP-9, which promotes the infiltration of inflammatory cells.^46^ These aberrant miRNA signatures may offer diagnostic biomarkers and therapeutic targets for CRVO.

#### 4.2.2 Transcription factor(TF)-miRNA co-regulation

The synergistic regulation of TF and miRNAs impacts retinal pathology through multiple mechanisms.^47^ At the molecular level, hypoxia-activated HIF-1α drives miRNA expression by binding to promoter regions, such as the HIF-1α/miR-210 axis.^48^ This high expression of TF and miRNAs is positively correlated (r ​≈ ​0.5, *P* ​< ​0.05) with vasoactive factors like VEGF and MCP-1, which may worsen CRVO associated with ME by triggering pathological neovascularization.^48^ At the regulatory network level, NF-КB activation induces miR-155 expression, enhancing IL-6 signaling by inhibiting SOCS1 and reactivating NF-КB, forming a pro-inflammatory feedback loop.^49^ These interactions involving HIF-1α/NF-КB and miR-210/miR-155 underpin retinal vascular leakage and injury by regulating angiogenesis, inflammation, and cellular stress.

#### 4.2.3 Regulatory role of long-chain non-coding RNAs (lncRNAs)

lncRNAs critically regulate retinal neovascularization (RNV) via epigenetic modulation, RNA-protein interactions, and competitive endogenous RNA networks.^50^ They significantly impact retinal diseases like diabetic retinopathy (DR) and retinopathy of prematurity (ROP), influencing key pathological processes: inflammation (*e.g.*, HOTAIR, SNHG16), apoptosis (MEG3, HOTTIP), and angiogenesis (MALAT1, MIAT, ANRIL).^51^ While MEG3 demonstrates protective effects, others (*e.g.*, MALAT1, HOTAIR) promote RNV.^52^, ^53^, ^54^

In ischemic RVO, characterized by pathological angiogenesis, blood-retinal barrier disruption, and ME.^55^ Crucially, lncRNAs coordinate the coupling between inflammation and angiogenic pathways in proliferative retinopathies.^54^ Although current research focuses on DR and ROP, the molecular similarities across diseases (*e.g.*, VEGF pathway activation, pericyte loss) offer clues to explore lncRNAs' role in RVO.^56^

## 5 Markers in RVO: from discovery to clinical utility

RVO diagnosis traditionally employs ophthalmological imaging (slit lamp, fundus photography, FFA). Advanced OCT/OCTA coupled with ML/DL algorithms now deliver quantitative imaging markers, enabling automated feature extraction for progression monitoring and treatment optimization.^57^ Complementarily, bioinformatics-driven analysis of systemic biomarkers (hematological, biochemical, molecular) identifies pathophysiological signatures. Integrated ML models predict gene-disease associations and novel therapeutic targets, facilitating precision risk stratification and targeted intervention.^58^

### 5.1 Structure and function assessment markers

#### 5.1.1 Imaging markers

**Fig. 1:**
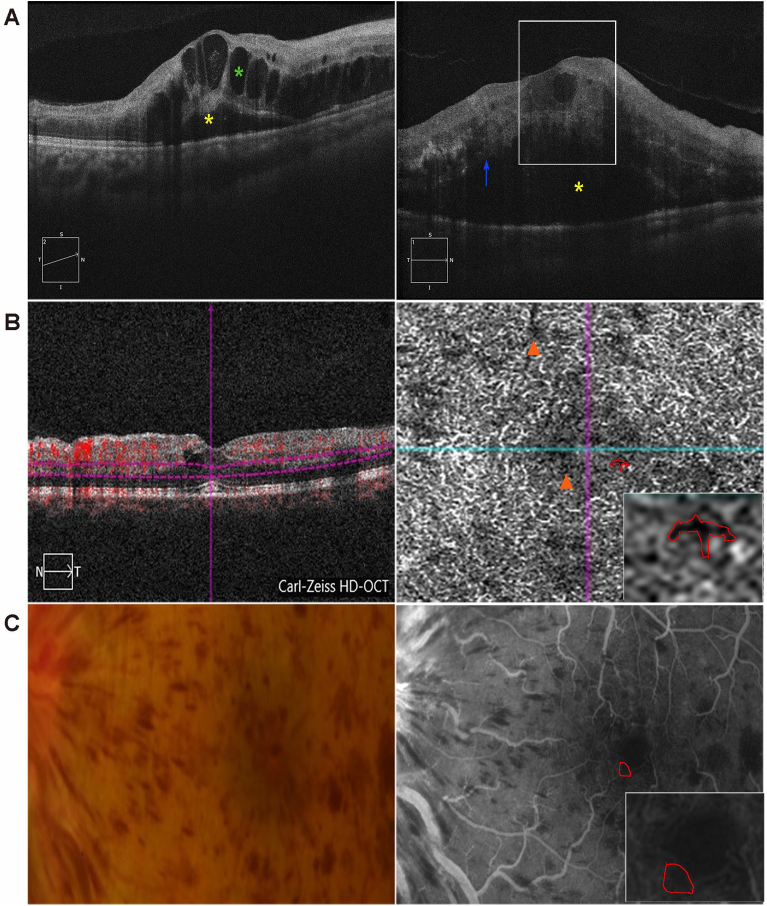
Multimodal Imaging Markers in Retinal Vein Occlusion: Integrated OCT, OCTA, and FFA Annotations Intraretinal Fluid (IRF, green asterisk in A), Subretinal Fluid (SRF, yellow asterisk in A), Disorganization of Retinal Inner Layers (DRIL, white border in A), Ellipsoid Zone disruption (EZ, blue arrow in A), External Limiting Membrane disruption (ELM, blue arrow in A); Deep Vessel Density (DVD, orange triangle in B), Foveal Avascular Zone (FAZ, red topographic map in B & C).

The key imaging markers discussed in this section are annotated in Fig. 1, with representative OCT, OCTA, and FFA images.

OCT enables high-resolution visualization of retinal microstructure, facilitating the identification of key markers relevant to RVO progression and treatment response. For fluid markers, reduced intraretinal fluid (IRF) volume correlates directly with improved visual acuity. A 100 ​nL decrease in foveal IRF corresponds to a mean gain of +2.13 letters. Similarly, subretinal fluid (SRF) reduction correlates more strongly, with each 100 ​nL decrease resulting in a mean gain of +5.88 letters.^59^ Concerning structural integrity, disruption of the EZ, often occurring alongside external limiting membrane (ELM) abnormalities, strongly influences visual prognosis; EZ disruption specifically associates with adverse visual outcomes (r ​= ​0.395, *P* ​= ​0.013).^60^ Disorganization of the DRIL, reflecting ischemic injury and ganglion cell degeneration, positively correlates with the final BCVA (Coefficient ​= ​0.588, *P* ​< ​0.001).^61^ For therapeutic monitoring, quantitative changes in CRT serve as a valuable metric for assessing anti-VEGF therapy efficacy, demonstrating good predictive value for treatment response (AUC: 0.745, Sensitivity: 63.4%; Specificity: 77.3%).^62^ Collectively, these quantitative OCT-derived metrics – encompassing fluid dynamics, structural integrity, and therapeutic response – provide an objective framework for understanding RVO pathophysiology and optimizing clinical management.

OCTA provides a non-invasive, quantifiable assessment of retinal microvasculature, identifying critical markers for RVO. For ischemic CRVO diagnosis, deep vessel density (DVD) ≤38.4% demonstrates superior accuracy (AUC ​= ​0.962, Sensitivity ​= ​100%, Specificity ​= ​92.3%), outperforming other parameters, including deep parafoveal vessel density (SPFVD, DPFVD) metrics.^63^ In ischemic BRVO with ME, baseline foveal avascular zone (FAZ) parameters (area, a-circularity index [AI]) predict anti-VEGF outcomes: larger FAZ area correlates with poorer final visual gain (FVG) (r ​= ​−0.701, *P* ​< ​0.05) and increased injection frequency (r ​= ​0.856, *P* ​< ​0.05). Progressive FAZ enlargement (area: 0.38 ​± ​0.02 ​→ ​0.39 ​± ​0.02 ​mm^2^; AI: 1.27 ​± ​0.02 ​→ ​1.31 ​± ​0.01) at 6 months associates with worse visual recovery, while higher peri-FAZ vessel density (FD-300) correlates with better FVG (r ​= ​0.537, *P* ​< ​0.05).^64^

FFA retains clinical value for evaluating vascular pathology, particularly in detecting leakage, demarcating non-perfusion zones, and differentiating ischemic subtypes. Evidence suggests intravitreal ranibizumab may contribute to transient macular ischemia, with short-term FAZ expansion observed in ischemic CRVO (0.69 ​± ​0.21 ​mm^2^ → 0.76 ​± ​0.25 ​mm^2^; r ​= ​0.286, *P* ​< ​0.001) and BRVO (0.61 ​± ​0.22 ​mm^2^ → 0.67 ​± ​0.31 ​mm^2^; r ​= ​0.180, *P* ​< ​0.05), correlating with reduced visual gain. Concomitant retinal vein constriction (*e.g.*, ischemic BRVO: r ​= ​−0.310, *P* ​< ​0.001) mirrored these changes, exhibiting partial recovery at 24 months.^65^ Despite limitations including contrast allergy risk, FFA provides essential dynamic functional assessment of vascular pathology. Combined with OCTA's structural metrics, this enables comprehensive evaluation of disease severity and prognosis.^66^

#### 5.1.2 Retinal electrophysiological markers

Multifocal electroretinography (mfERG) offers high-resolution quantification of regional retinal function, making it particularly valuable in the assessment of RVO.^67^ In CRVO, affected eyes demonstrate significantly reduced a-wave/b-wave/PhNR amplitudes (*P* ​< ​0.001) and prolonged latencies versus fellow eyes, with PhNR amplitude strongly correlating with central foveal thickness (r ​= ​−0.598, *P* ​= ​0.004) and greater reduction in ischemic subtypes.^68^ BRVO studies show similar amplitude/latency abnormalities (*P* ​< ​0.05), where PhNR amplitude tracks foveal thickness (r ​= ​−0.465, *P* ​= ​0.007) and improves with edema resolution (PhNR: *P* ​= ​0.033).^69^ mfERG precisely maps functional recovery, evidenced by 43–68% amplitude increases (N1/P1 waves) in bevacizumab-treated BRVO quadrants (Q1-Q2; *P* ​< ​0.05), while revealing post-treatment dissociation from structural improvements.^70^

### 5.2 Circulatory system and molecular network biomarkers in RVO

Key systemic and molecular biomarkers, along with their associated pathomechanisms and functions, are comprehensively summarized in Table 1.

**Table 1:** Circulatory system and molecular network biomarkers in RVO: Pathomechanisms and functions.

Year	Reference	Study Type/Sample	Key Molecular Targets/Pathways	Main Results/Conclusions
2020	Wei et al.^32^	Human study: AH (n ​= ​35, CRVO vs. cataract ​= ​15 vs. 20)	Glutamate ↓, Glutamine ↓; Lactate ↑, Glutamine ↑ (after anti-VEGF)	Glutamate ↓ (4.26 vs. 6.89 ​μg/ml, *t* ​= ​3.053, *P* ​= ​0.009), Glutamine ↓ (10.96 vs. 18.35 ​μg/ml, *t* ​= ​2.525, *P* ​= ​0.017); Lactate & Glucose negatively correlated with RGC thickness after anti-VEGF injection(r ​= ​−0.619, *P* ​= ​0.024; r ​= ​−0.754, *P* ​= ​0.003, respectively).
2014	Minniti et al.^33^	Human study: plasma (n ​= ​162, CRVO vs. BRVO vs. controls ​= ​47 vs. 44 vs. 71)	Homocysteine ↑; Vitamin B12 ↓	CRVO: Homocysteine ↑ (9.5 vs. 8.1 ​μM, *P* ​= ​0.05); OR ​= ​1.20 (95% CI 1.02–1.41; *P* ​= ​0.03); Vitamin B12 ↓ (422 vs. 505 ​pg/mL, *P* ​= ​0.02); OR ​= ​0.997 (95% CI 0.99–1.0; *P* ​= ​0.008); BRVO: Homocysteine ↑ (8.9 vs. 8.1 ​μM, *P* ​> ​0.05)
2023	Doğan et al.^71^	Human study: blood (n ​= ​60, RVO-SMD vs. RVO ​= ​30 vs. 30)	Neutrophil levels ↑, NLR ↑, SII ↑	NLR & SII in diagnosing RVO-SMD: AUC 0.714 & 0.681, Sens 73% & 63%, Spec 63% & 63%, respectively.
2023	Timur et al.^72^	Human study: blood (n ​= ​180, RVO-SRD vs. RVO vs. controls ​= ​60 vs. 60 vs. 60)	SII ↑, NLR ↑	NLR & SII in diagnosing RVO-SRD: AUC 0.707 & 0.765, Sens 66.7% & 68.3%, Spec 65% & 68.3%, respectively; NLR positively correlated with CRT (r ​= ​0.417, *P* < 0.001).
2021	Wan et al.^73^	Human study: plasma (n ​= ​125, RVO vs. control ​= ​77 vs. 48)	NETs markers (cfDNA & MPO-DNA & H3Cit) ↑	cfDNA & MPO-DNA & H3Cit in diagnosing RVO: AUC 0.859 & 0.871 & 0.928, respectively.
2017	Guclu et al.^74^	Human study: blood (n ​= ​166, CRVO vs. BRVO vs. controls ​= ​44 vs. 68 vs. 54)	FAR ↑	FAR & fibrinogen positively correlated with ischemia RVO (r ​= ​0.732, *P* ​= ​0.001; r ​= ​0.669, *P* ​= ​0.001, respectively).
2016	Yilmaz et al.^75^	Human study: plasma (n ​= ​168, RVO vs. control ​= ​83 vs. 85)	MPV ↑, PDW ↑, PLCR ↑	RVO: MPV ↑ (8.26 vs.7.41 ​fL, *P* ​= ​0.006); PDW ↑ (13.43 vs. 12.19%, *P* ​= ​0.002); and PLCR ↑ (30.62 vs. 28.59%, *P* ​= ​0.003).
2020	Kurtu et al.^76^	Human study: blood (n ​= ​64, RVO vs. control ​= ​32 vs. 32)	PLR ↑	PLR in diagnosing RVO: AUC 0.726, Sens 69%, Spec 72%; Greater retinal ischemia.
2018	Ozkok et al.^77^	Human study: blood (n ​= ​193, BRVO vs. CRVO vs. controls ​= ​70 vs. 56 vs. 67)	RDW ↑	RDW correlated with initial BCVA and final BCVA (r ​= ​0.443, *P* ​< ​0.0001; r ​= ​0.379, *P* ​< ​0.0001, respectively).
2022	Kazantzis et al.^78^	Human study: plasma (n ​= ​108, RVO vs. control ​= ​54 vs. 54)	NLR ↑, RDW ↑, MPV ↑, SII ↑	NLR & RDW & MPV & SII in diagnosing RVO: AUC 0.648 & 0.645 & 0.618 & 0.640, respectively; Sens 46.2% & 61.5% & 67.3% & 55.8%, respectively; Spec 77.8% & 59.3% & 57.4% & 68.5%, respectively.
2012	Bharathi et al.^79^	Human study: plasma (n ​= ​77, RVO vs. control ​= ​23 vs. 54); *In vitro* (BREC cells)	Oxidative Stress Markers (TBARS↑, SOD↓, TAC↓), Hcy ↑	TAC & TBARS negatively correlated (*P* < 0.001); Hcy & TBARS positively correlated (*P* ​= ​0.029); Hcy ↑ TBARS in vitro → CRVO oxidative stress.
2024	Deng et al.^81^	Human study: plasma (n ​= ​60, RVO vs. control ​= ​30 vs. 30); *In vivo* (RVO mouse model)	NETs Pathway (cf-DNA↑, MPO-DNA↑, NE↑)	NETs ↑ in human/mouse RVO ​+ ​thrombi; DNase I diminished NETs formation and reduced thrombus duration → RVO protection.
2014	Tuuminen et al.^83^	Human study: vitreous fluid (n ​= ​44, RVO vs. macular hole ​= ​4 vs. 40)	Fibrotic Pathway (TGF-β1↑, MMP-9↑)	Ischemic RVO: TGF-β1↑ (92.0 vs. 18.3 ​pg/ml, *P* ​= ​0.002) & MMP-9↑ (847.9 vs. 87.7 AU/ml, *P* ​= ​0.010)→ Greater retinal ischemia
2014	Yasuda et al.^84^	Human study: AH (n ​= ​28, hCRVO vs. BRVO ​= ​12 vs. 16)	VEGF ↑	hCRVO vs. BRVO: VEGF↑ (504 vs. 148 ​pg/ml, *P* ​< ​0.05) & ERG delay (33.5 vs. 29.8 ​ms, *P* ​< ​0.01)→ Greater retinal ischemia
2014	Noma et al.^85^	Human study: AH (n ​= ​40, BRVO vs. cataract ​= ​31 vs. 9)	Growth factors (VEGF/PlGF/PDGF-AA) ↑; VEGFRs (sVEGFR-1/-2) ↑; Inflammatory (sICAM-1/MCP-1/IL-8) ↑	sVEGFR-1/2 correlated with growth/inflam. factors; SRT correlated with VEGFRs & inflam.; Anti-VEGFR-1/2 therapy potential for BRVO-ME & SRT.
2015	Shchuko et al.^98^	Human study: AH (n ​= ​64, CRVO vs. BRVO vs. controls ​= ​18 vs. 26 vs. 20)	VEGF/IL-6/IL-8/IL-10/IL-12p70/IL-13/IL-15/MCP-1 ↑ RAIL-1/IL-9/RANTES ↓	Differential CRVO/BRVO patterns, CRVO vs. BRVO: CRVO dominant: VEGF/IL-6/IL-8/IL-10/IL-12p70/IL-13/MCP-1 ↑, RANTES ↓; CRVO & BRVO: RAIL-1/IL-9/↓, IL-15 ↑.

**Abbreviations**: AH, Aqueous humor; AUC, The area under the curve; Sens, Sensitivity; Spec, Specificity; CRT, Central retinal thickness; GC-IPL, Ganglion cell-inner plexiform layer; SRT, Serous retinal thickness; CMT, Central macular thickness; RGC, retinal ganglion cells; ERG, Electroretinogram; hCRVO, Hemicentral retinal vein occlusion; SMD, Serous macular detachment; NLR, Neutrophil-lymphocyte ratio; SII, Systemic immune-inflammation index; SRD, Serous retinal detachment; PLR, Platelet-to-lymphocyte ratio; NETs, Neutrophil extracellular traps; FAR, Fibrinogen to albumin ratios; PLCR, Platelet large cell ratio; BREC, Bovine retinal endothelial cells; CFT, Central foveal thickness.

#### 5.2.1 Systemic circulatory dysregulation: the triad of inflammation, thrombosis, and oxidative stress

Key inflammatory indices show diagnostic utility: systemic inflammation emerges as a key contributor to RVO pathogenesis, evidenced by elevated NLR (AUC ​= ​0.714, Sensitivity ​= ​73%, Specificity ​= ​63%) and systemic immune-inflammatory index (SII: AUC ​= ​0.681, Sensitivity ​= ​63%, Specificity ​= ​63%).^71^ These indices correlate with disease severity, where NLR positively associates with CRT (r ​= ​0.417, *P* ​< ​0.001).^72^ Neutrophil extracellular traps (NETs) biomarkers demonstrate significant diagnostic value in RVO (cfDNA/MPO-DNA/H3Cit: AUC ​= ​0.859–0.928) and drive vascular injury through pro-inflammatory mediator release.^73^

Prothrombotic alterations further characterize RVO, with the fibrinogen-albumin ratio (FAR) strongly correlating with retinal ischemia (r ​= ​0.732, *P* ​= ​0.001), reflecting hypercoagulable microthrombosis.^74^ Platelet hyperactivity indicated by increased mean platelet volume (MPV: 8.26 vs. 7.41 ​fL, *P* ​= ​0.006) and distribution width (PDW: 13.43% vs. 12.19%, *P* ​= ​0.002), with platelet-lymphocyte ratio (PLR) demonstrating diagnostic utility (AUC ​= ​0.726, Sensitivity ​= ​69%, Specificity ​= ​72%) for severe ischemia.^75^^,^^76^ Erythrocyte rheological abnormalities reflected by elevated RDW correlate with poor visual outcomes (r ​= ​0.379, *P* ​< ​0.0001) and demonstrate diagnostic potential (AUC ​= ​0.645).^77^^,^^78^

Oxidative stress integrates inflammatory and thrombotic pathways, manifesting as depleted antioxidants (TAC↓, SOD↓) and NETs-mediated endothelial dysfunction.^79^ This endothelial dysfunction may affect vascular permeability and also lead to persistent damage to the retinal microvasculature, further aggravating the pathological process of RVO.^80^ Experimental evidence confirms elevated NETs formation in both human RVO thrombi and murine models, where DNase I administration reduced thrombus duration (*P* ​< ​0.05) by diminishing NETosis.^81^ This triad of dysregulation provides actionable biomarkers for mechanistic stratification and targeted therapeutic development.

#### 5.2.2 Molecular drivers and signaling networks in retinal pathogenesis

##### 5.2.2.1 VEGF dynamics in RVO pathogenesis

VEGF-A serves as the principal regulator of pathological angiogenesis in RVO.^82^ Vitreous fluid analysis confirms significant VEGF elevation in ischemic RVO, establishing its central angiogenic role.^80^^,^^83^ This dysregulation exhibits subtype-specific severity, where AH analysis reveals markedly higher VEGF concentrations in CRVO compared to BRVO (504 vs. 148 ​pg/mL, *P* ​< ​0.05), correlating with ME severity.^84^^,^^85^ Mechanistically, VEGF-A activates PI3K/AKT and MAPK/ERK pathways via VEGFR-1/2 receptors, driving endothelial proliferation and vascular permeability.^86^ Hypoxia-inflammation-VEGF feedback loops further potentiate retinal damage.^87^ Anti-VEGF therapy (*e.g.*, ranibizumab) effectively reduces AH VEGF levels and improves vision.^88^ However, activation of bypass signals (*e.g.*, Ang-2/Tie2) can lead to drug resistance, so dual Ang-2/VEGF-A inhibition therapy may improve efficacy.^89^

##### 5.2.2.2 Proteolytic remodeling by MMPs

MMPs exhibit dual roles in vascular remodeling and inflammation amplification. Vitreous MMP-9 levels significantly elevate in RVO (847.9 vs. 87.7 AU/mL in controls, *P* ​= ​0.010), forming an IL-6/STAT3-driven feedback loop.^83^^,^^90^ MMP-9 activates IL-6 and TLR4/NF-κB pathways, while MMP-2 degrades basement membrane collagen IV and tight junction proteins (occludin/claudin-5), compromising blood-retinal barrier integrity.^91^, ^92^, ^93^, ^94^, ^95^ Notably, clinical data have shown that elevated levels of MMPs in the AH of RVO patients are negatively correlated with visual improvement after anti-VEGF treatment. Still, translational clinical trials of broad-spectrum MMP inhibitors (*e.g.*, marimastat) or MMP-9 specific antibodies (*e.g.*, GS-5745) are in the exploratory phase, and their safety and efficacy need to be further validated.^96^^,^^97^

##### 5.2.2.3 Inflammatory cytokine networks and subtype heterogeneity

Beyond VEGF and MMPs, dysregulated cytokine networks critically orchestrate retinal inflammation in RVO. Research evidence suggests that CRVO elevations in pro-inflammatory (IL-1β, IL-6, IL-8, IL-12p70, IL-13), and chemotactic mediators (MCP-1, CXCL-10, CCL2), alongside immune modulators (IL-10, TGF-β) and acute-phase proteins (SAA). Conversely, BRVO demonstrates prominent increases in Th1/Th17-associated cytokines (IL-12, IL-15, IL-17), soluble adhesion molecules (sICAM-1), with shared elevations in TGF-β, and SAA.^98^, ^99^, ^100^

Mechanistically, IL-6 drives NF-КB-dependent retinal inflammation by activating the JAK/MAPK pathway, while IL-8 induces VEGF expression and promotes neovascularization.^101^, ^102^, ^103^ Clinically, inhibiting the IL-6 pathway presents a promising strategy for anti-VEGF-resistant cases. Early evidence suggests that tocilizumab, an IL-6 receptor antagonist, may reduce ME in refractory RVO and uveitis by disrupting IL-6 trans-signaling.^104^

#### 5.2.3 Interplay between systemic and local mechanisms

Systemic circulatory dysregulation—characterized by inflammation, hypercoagulability, and oxidative stress—synergistically amplifies retinal damage in RVO through dynamic crosstalk with local molecular pathways.^105^ Activated blood components (*e.g.*, neutrophils, platelets, NETs, and oxidative byproducts) disrupt endothelial integrity, thereby potentiating the activity of intraocular mediators such as VEGF, MMPs, and inflammatory cytokines.^80^

Integrating systemic biomarker profiles (blood-based indices) with local molecular signatures (vitreous and AH biomarkers) enables comprehensive pathophysiological mapping. This method may reveal subtype-specific mechanisms (*e.g.*, CRVO vs. BRVO) and highlight potential targets for tailored interventions, improving diagnostic and therapeutic precision in RVO management.

## 6 Challenges in research and translation

This review synthesizes how integrating ML and bioinformatics with multimodal data holds significant translational promise for RVO. By enabling predictive modeling for prognosis and treatment response, elucidating complex molecular pathways underlying pathogenesis, and identifying novel diagnostic and prognostic biomarkers, these integrated approaches offer a pathway towards personalized risk assessment, earlier intervention, and the development of targeted therapies. However, realizing this full translational potential faces substantial hurdles. Critical barriers currently impede the effective clinical translation of insights derived from multimodal RVO studies. Key challenges include.

### 6.1 Data heterogeneity and integration challenges

A primary obstacle stems from the inherent heterogeneity of multidimensional data sources. These sources typically combine EMRs, medical imaging datasets (*e.g.*, OCT, FFA), and multi-omics profiles (*e.g.*, genomic, proteomic, metabolomic). The absence of standardized data formats and inconsistent annotation protocols across institutions complicates data harmonization. This variability introduces potential analytical biases and compromises the reliability of predictive models.^106^ For instance, disparities in OCT image acquisition parameters between clinical centers may systematically affect feature extraction in deep learning algorithms.

### 6.2 Model interpretability limitations

The limited transparency of advanced ML architectures presents a significant implementation barrier. Deep neural networks, while achieving superior classification accuracy in retinal image analysis, frequently operate as opaque "black box" systems.^107^ This opacity creates significant barriers to clinical adoption, as practitioners require transparent decision-making processes to validate diagnostic recommendations.

### 6.3 Translational validation deficits

Three critical validation barriers impede clinical translation of RVO research: First, Inconsistent biomarker standardization in multi-omics studies, particularly for metabolite quantification and proteomic normalization, hinders cross-study reproducibility. Second, there is a deficit in longitudinal validation. Few models are rigorously tested in multi-center trials with extended follow-up periods. Moreover, concerns about clinical relevance persist, as preclinical findings often fail to demonstrate clear therapeutic correlations, particularly in the modulation of angiogenic pathways.^108^^,^^109^

### 6.4 Ethical and data governance considerations

The use of sensitive patient data in multimodal RVO research raises critical ethical considerations. Pseudonymized datasets containing genetic information and high-resolution retinal images require robust cybersecurity frameworks to prevent re-identification risks. Current regulatory landscapes present additional complexities, as international data sharing - essential for adequate validation cohort sizes - must navigate conflicting privacy regulations.^110^

## 7 Future directions and research priorities

To bridge the translational gaps identified in multimodal RVO research, we propose the following prioritized research framework.

### 7.1 Multiscale phenotyping framework enhancement

To advance RVO subtyping precision, we propose integrating OCTA-derived metrics (*e.g.*, parafoveal vessel density, venous diameter ratio) with AH metabolomics (*e.g.*, lactate, glutamine levels) through transformer-boosted gradient machine frameworks. This integrated approach may enhance model interpretability by capturing microvascular alterations and systemic metabolic dysregulation while striving to preserve diagnostic accuracy.^111^ Implementation requires: 1) Prospective multicenter validation trials employing standardized protocols such as 6 ​× ​6 ​mm OCTA scan patterns with fixed resolution and unified LC-MS/MS platforms for metabolite quantification; and 2) Establishment of international consortium agreements to develop cross-institutional metadata frameworks, enable mutual recognition of ethical approvals, and harmonize technical parameters for enhanced interoperability.

### 7.2 Standardized data infrastructure development

Establishing standardized data formats and annotation protocols across institutions is crucial to address data heterogeneity. The development of centralized data repositories with robust data governance frameworks can facilitate data harmonization and sharing. These repositories should incorporate metadata standards that account for imaging acquisition parameters, clinical annotations, and multi-omics data processing details. Implementation of federated learning approaches can enable model training across distributed datasets without compromising data privacy, thereby reducing analytical biases and enhancing model reliability.^112^

### 7.3 Interpretable model design and validation

Future research should focus on developing interpretable ML architectures that balance accuracy and transparency.^113^ Techniques such as attention mechanisms, saliency mapping, and rule extraction can be employed to shed light on the decision-making processes of complex models. Validation of these interpretable models through clinician feedback loops and simulation studies can help bridge the gap between model performance and clinical acceptance. Creating benchmark datasets with Ground Truth annotations will provide a foundation for evaluating both model accuracy and interpretability in a clinically meaningful context.^114^

### 7.4 Ethical frameworks for data utilization

Given the ethical considerations surrounding sensitive patient data, there is a pressing need for comprehensive ethical frameworks. These frameworks should outline principles for data anonymization, consent management, and secure data sharing. Collaboration with regulatory bodies can help harmonize privacy regulations across jurisdictions, facilitating international research collaborations.^115^^,^^116^ Patient engagement initiatives can ensure that data utilization aligns with patient values and expectations, fostering trust in RVO research endeavors.

## 8 Conclusions

In summary, the evolving synergy of ML and bioinformatics is promoting RVO research from static, single-dimensional analyses to a dynamic, multidimensional paradigm. Addressing these priorities requires coordinated efforts in data standardization, regulatory alignment, and translational validation, ultimately enabling personalized therapeutic strategies grounded in multimodal data convergence.

## Study approval

This review article synthesizes data from published literature. The clinical images in Fig. 1 originated from a retrospective cohort at the First Affiliated Hospital of Jinan University. Their usage was approved by the Institutional Ethics Committee (Approval No. KY-2024-074) under the principles of the Declaration of Helsinki.

## Author contributions

The authors confirm contribution to the paper as follows: Chunlan Liang drafted the primary manuscript. Lian Liu and Jingxiang Zhong provided suggestions and revised the manuscript for final submission. All authors reviewed the results and approved the final version of the manuscript.

## Funding

This work was supported by the National Natural Science Foundation of China (82271094 to J.Z.).

## Declaration of competing interest

The authors declare that they have no known competing financial interests or personal relationships that could have appeared to influence the work reported in this paper.

## Abbreviations

RVO: Retinal Vein Occlusion
ML: Machine Learning
DL: Deep Learning
EMRs: Electronic Medical Records
AUC: Area Under the Curve
NLR: Neutrophil-Lymphocyte Ratio
SBP: Systolic Blood Pressure
ME: Macular Edema
BCVA: Best-Corrected Visual Acuity
EZ: Ellipsoid Zone
CRT: Central Retinal Thickness
DRIL: Disorganization of Retinal Inner Layers
CNN: Convolutional Neural Network
FFA: Fundus Fluorescein Angiography
LA-Net: Layered Attention Network
GAN: Generative Adversarial Network
DRW: Dynamic Risk Warning
AMD: Age-Related Macular Degeneration
OCTA: Optical Coherence Tomography Angiography
AH: Aqueous Humor
CMT: Central Macular Thickness
RGC: Retinal Ganglion Cell
miRNA: MicroRNAs
mRNA: messenger RNA
TF: Transcription Factor
lncRNA: Long Non-Coding RNA
RNV: Retinal Neovascularization
DR: Diabetic Retinopathy
ROP: Retinopathy of Prematurity
IRF: Intraretinal Fluid
SRF: Subretinal Fluid
ELM: External Limiting Membrane
DVD: Deep Vessel Density
FAZ: Foveal Avascular Zone
AI: A-circularity Index
FVG: Final Visual Gain
mfERG: Multifocal Electroretinogram
SII: Systemic Immune-Inflammation Index
NETs: Neutrophil Extracellular Traps
FAR: Fibrinogen-Albumin Ratio
PLR: Platelet-Lymphocyte Ratio
